# Characterization of Carbapenemases in Extensively Drug Resistance *Acinetobacter baumannii* in a Burn Care Center in Iran

**Published:** 2015

**Authors:** Leila Azimi, Malihe Talebi, Mohammad-Reza Pourshafie, Parviz Owlia, Abdolaziz Rastegar Lari

**Affiliations:** 1 *Antimicrobial Resistance Research Center, Iran University of Medical Sciences, Tehran, Iran.*; 2 *Department of Microbiology, Iran University of Medical Sciences, Tehran, Iran.*; 3 *Department of Bacteriology, Pasteur Institute of Iran, Tehran, Iran.*; 4 *Molecular Microbiology Research Center, Shahed University, Tehran, Iran.*

**Keywords:** *Acinetobacter baumannii*, carbapenemases, KPC

## Abstract

The emergence of multidrug resistance (MDR) and extensively drug resistance (XDR) in *Acinetobacter baumannii *has made an important challenge in the treatment of infections caused by this organism. The ability of carbapenemase production is one of the main mechanisms for the emergence of MDR and/or XDR in *A. baumannii*. The aim of this study was to detect carbapenemase producer *A. baumannii*. In this study, 65 imipenem resistant* A. baumannii *were collected from burned patients. Biochemical identification, antibiotic susceptibility test and multiplex polymerase chain reactions for the detection of carbapenemases genes were performed. The results showed that all strains carried *bla*_OXA-51_. 83%, 12.5% and 9.23% strains harbored *bla*_OXA-23_, *bla*_VIM_ and *bla*_KPC_ genes, respectively. None of the isolates carried *bla*_IMP_, *bla*_OXA-48_, bla_NDM-1 _and *bla*_SPM-1_ genes. The results of this study indicate the emergence of *Klebsiella pneumoniae* Carbapenemase (KPC) in *A. baumannii* causing nosocomial infections in burned patients which can be important for hospital infection prevention systems in Iran.

*Acinetobacter baumannii *has been recognized as one of the important causes of nosocomial infections in hospitalized patients, particularly in burned ones in recent years (1- 3). During the past decade, in some countries, this opportunistic pathogen had high rate of infection in burned patients and was reported to be the second most common cause of nosocomial infections in burned patients (1, 2, 4-7). *A. baumannii *has been shown to acquire fast antibiotic resistance elements (8, 9). Recently, this Gram negative bacilli has shown resistance to the most available antibiotics followed by the emergence of multiple (MDR) and extensive drug resistance (XDR) strains (2, 8-10). This has partly been due to the extensive use of broad spectrum antibiotics especially in burned patients (9). Carbapenems are generally used for antibiotic therapy in infections due to MDR* A. baumannii*. However, the emergence of carbapenem resistant isolates has made it difficult to treat such infections (2, 3, 8, 11-14). One of the most common and important mechanisms in carbapenem resistance in *A. baumannii *is their ability to produce carbapenemase enzymes (2, 3, 6-8). Among the carbapenem hydrolyzing β-lactamases, class D oxacillinases (OXA type) is the most prevalent in *A. baumannii* strains (3, 9, 14). On the other hand, some studies have reported class B beta lactamases (metallo- beta lactamases) in *A. baumannii *as the most prevalent carbapenemase after OXA- type (8). The significant importance is the worldwide prevalence of *Klebsiella pneumoniae* Carbapenemase (KPC) producers (15, 16). Gram negative KPC producers can be resistant to all beta lactam antibiotics except aztreonam (17). Most of these carbapenemases have been located in transferable genetic elements and can spread among *A. baumannii *and even into other Gram- negative bacilli (2, 8). Therefore, the determination of carbapenemase- producing strains in healthcare systems could help in the eradication of *A. baumannii *antibiotic resistant strains.

**Table 1:** Sequence of primers

**Primer**	**Sequence (5** ^'^ **→3** ^'^ **)**	**PCR pdoduct size (bp)**	**References**
VIM F	TTGACACTCCATTTACDG	390	
VIM R	GATYGAGAATTAAGCCACYCT		
Imp F	GATGGTGTTTGGTCGCATA	139	8
Imp R	CGAATGCGCAGCACCAG		
OXA-23 F	GATGTGTCATAGTATTCGTCGT	1050	
OXA-23 R	TCACAACAACTAAAAGCACTGT		9
OXA-48-ike F	CCAAGCATTTTTACCCGCATCKACC	389	
OXA-48-like R	GYT TGA CCA TAC GCT GRC TGC G		
NDM-1 F	CCCGGCCACACCAGTGACA	129	
NDM-1 R	GTAGTGCTCAGTGTCGGCAT		9
SPM-1 F	GGGTGGCTAAGACTATGAAGCC	447	
SPM-1 R	GCCGCCGAGCTGAATCGG		
OXA-51-like F	TAATGCTTTGATCGGCCTTG	353	7
OXA-51-like R	TGGATTGCACTTCATCTTGG		
KPC F	GTATCGCCGTCTAGTTCTGC	636	18
KPC R	GGTCGTGTTTCCCTTTAGCC		

**Table 2:** PCR conditions for bla genes amplification

**No. Cycles**	**Final extension Temperature (time)**	**Extension Temperature (time)**	**Annealing Temperature (time)**	**Denaturation** **Temperature (time)**	**Initial denaturation Temperature (time)**	**Gene**
30	72 ^o^C (7 min)	72 ^o^C (1 min)	60 ^o^C (40 sec)	94 ^o^C (40 sec)	94 ^o^C (10 min)	*bla*_VIM _& *bla*_IM: _
30	72 ^o^C (7 min)	72 ^o^C (1 min)	55^ o^C (1 min)	94 ^o^C (30 sec)	94 ^o^C (1 min)	*bla*_OXA-23_ & *bla*_OXA-48_
30	72 ^o^C (1 min)	72 ^o^C (1 min)	60 ^o^C (40 sec)	94 ^o^C (30 sec)	94 ^o^C (1 min)	*bla*_NDM-1_ & *bla*_SPM-1_
30	72 ^o^C (5 min)	72 ^o^C (1 min)	56^ o^C (1 min)	94 ^o^C (1 min)	94 ^o^C (5 min)	*bla*_KPC_
30	72 ^o^C (5 min)	72 ^o^C (1 min)	58^ o^C (1 min)	94 ^o^C (45 sec)	94 ^o^C (5 min)	*bla*_OXA-51_

## Materials and methods

**Bacterial isolates**

This cross-sectional study was conducted in Motahari Hospital which is the only referral burn center in Tehran, Iran. Sixty-five imipenem resistant *A. baumannii* isolated from burn wound were collected from admitted patients from April to July 2013. Primary identification of these strains was performed by conventional biochemical tests such as oxidase, TSI, SIM and growing at 44 ^o^C as *A. baumannii*. The identification of isolates was confirmed by *API* 20 NE (Bio-Merieux, Lyon, France) strip test.

**Antimicrobial susceptibility testing**

The antibiotic susceptibility testing was performed by disc diffusion method on Mueller–Hinton agar using cefotaxime (30 µg), ceftazidime (30 µg), imipenem (10 µg), ticarcillin (75 µg), ticarcillin- clavulanic acid (75/10 µg), piperacillin (100 µg), piperacillin- tazobactam (100/10 µg), ciprofloxacin (5 µg), gentamicin (10 µg), tobramycin (10 µg), amikacin (30 µg), tetracycline (30 µg), trimethoprim (5 μg) and trimethoprim- sulfamethoxazole (1.25/23.75 μg) according to clinical and laboratory standards institute (CLSI) 2011. The standard antibiotic disks used in this study were from MAST Company (Mast Diagnostics, UK). *P. aeruginosa* ATCC 27853 was used as control strain in the antibiotic susceptibility testing. Detection of extended spectrum beta lactamase (ESBL) has been conducted by combination disc method by using ceftazidime and ceftazidime plus clavulanic acid. Strains with increasing at least 5 mm in the diameter of inhibition zone around ceftazidime plus clavulanic acid in contrast of ceftazidime alone were considered as an ESBL producer.

**PCR amplification for carbapenemases produ-ction genes**

Extraction of bacterial DNA was performed with a plasmid Minikit (Qiagen GmbH, Hilden, Germany) according to the manufacturer’s instructions.

Three different sets of multiplex PCR for* bla*_VIM_, *bla*_IMP_ and *bla*_OXA-23_, *bla*_OXA-48_ and *bla*_NDM-1_, *bla*_SPM-1_ genes and common PCR for *bla*_KPC_ and *bla*_OXA-51_genes were designed. The lists of primers used are shown in Table 1 and amplifivation conditions are represented in Table 2.

**Table 3:** Antibiotic resistance patterns

**patterns**	**Antibiotic Resistance patterns**	**Number**	**Percentage**
**1**	CTX CAZ CEF IMI PTZ PYR TC TC-C GM AK TO TM SXT CI	36	55
**2**	CTX CAZ CEF IMI PTZ PYR TC TC-C GM AK TO TM SXT CI T	16	25
**3**	CTX CAZ CEF IMI PTZ PYR TC TC-C AK TM SXT CI T	7	11
**4**	CTX CAZ CEF IMI PTZ PYR TC TC-C GM TO TM SXT CI	3	5
**5**	CTX CAZ CEF IMI PTZ PYR TC TC-C AK TO TM SXT CI T	2	3
**7**	CTX CAZ CEF IMI PTZ PYR TC TC-C AK TO TM SXT CI	1	1

CTX: cefotaxime, CAZ: ceftazidime, CEF: CEFEPIME, IMI: imipenem, PTZ: piperacillin-Tazobactam, PRL: piperacillin, TC: ticarcillin, TC-c: ticarcillin- clavulanic acid, GM: gentamicin, AK: amikacin, TO: tobramycin, TM: trimethoprim, SXT: trimethoprim- sulfamexazole, CI: ciprofloxacin, T: tetracycline

**Table 4:** Number and percentages of detected *bla* genes

***bla***_VIM_ ** and ** ***bla***_OXA_23_	***bla***_KPC_ ** and ** ***bla***_OXA_23_	***bla***_VIM_	***bla***_OXA_23_	***bla***_OXA-51_	**genes**
6(9.52%)	6(9.52%)	2(3.1%)	42(66.6%)	63(100%)	NO.(%)

PCR products were analyzed by electrophoresis on 1.5% agarose gel with SYBR safe staining. Direct sequencing of amplicons was carried out using ABI 3730X capillary sequencer (Genfanavaran, Macrogen, Seoul, Korea).

## Results

A total of 65 *A. baumannii *were isolated from 65 hospitalized burned patients, 15 (23%) females and 50 (77%) males. The age of patients were between 9 months to 72 years and total body burned surface area (TBSA) was more than 6%. Causes of burn were classified as follows: fire 6 (11%), hot water 6 (11%), electricity 10 (17%) gas 10 (17%), 6 (11%) chemical materials and others 19 (33%). Based on antibiotic susceptibility test, 97% of strains were resistant to all tested beta lactam antibiotics and 80% of them showed resistance to all tested aminoglycoside antibiotics.

The results of antibiotic susceptibility tests indicated seven antibiotic resistance patterns (Table 3). Pattern one was observed in 55% of isolates. Those strains were resistant to all tested antibiotics except colistin and tetracycline. In pattern two, all strains were resistant to the tested antibiotics which were observed in 25% of strains. In 20% strains, resistance to at least one of aminoglycoside antibiotics were observed according to antibiotic resistance patterns.

The Verona integron-encoded metallo-β-lactamase (VIM) and KPC producing strains were observed just in two antibiotic resistance patterns. OXA-23 and OXA-51 producing strains showed all seven antibiotic resistance patterns.

The ability of ESBL production was indicated just in one strain in combination disc method.

**Fig. 1:**
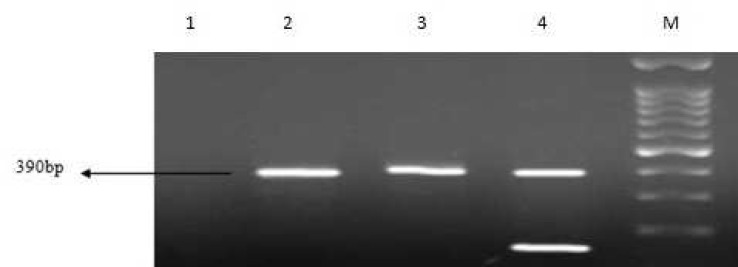
Multiplex PCR amplification fragments for the detection of *vim *and* imp *gene among *Acinetobacter baumannii* isolates. Lane 1: negative control, lanes 2, 3: positive *vim* strains, lane 4: positive control of *vim* and *imp *genes and M: 1kb DNA size marker

**Fig. 2:**
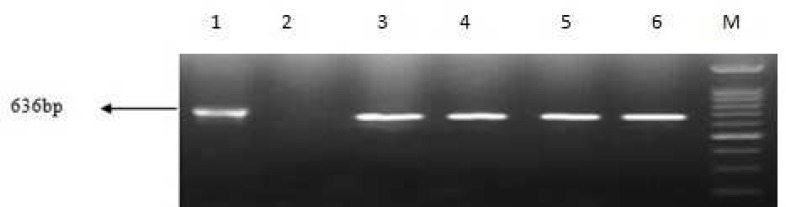
PCR amplification fragments for the detection of *kpc *gene among *Acinetobacter baumannii* isolates. Lane 1: positive control of *kpc* gene, lane 2: negative control, lanes 3- 6: positive *kpc* strains and M: 1kb DNA size marker

**Fig. 3:**
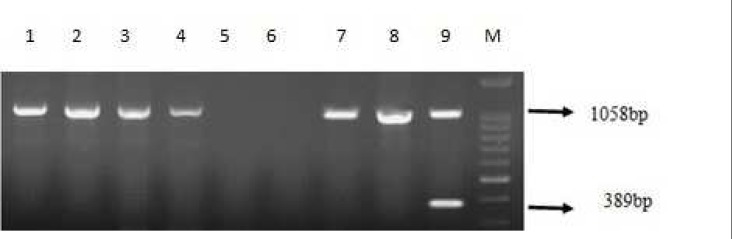
PCR amplification fragments for the detection of *oxa-23 *and* oxa-48 * genes among *Acinetobacter baumannii* isolates. Lane 1- 4 and 7, 8: positive oxa-23 strains, lanes 5, 6: negative control of *oxa-23 *and* oxa-48* genes*, *lane 9: positive control of *oxa-23 *and* oxa-48* genes and M: 1kb DNA size marker

**Fig 4:**
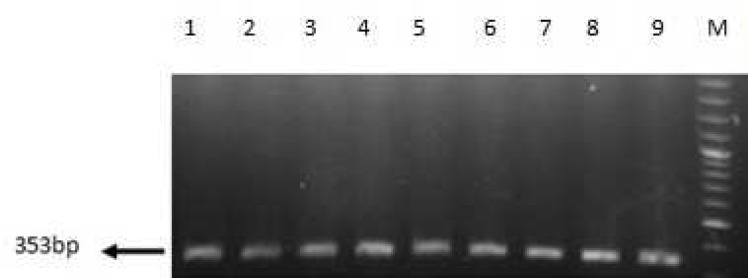
PCR amplification fragments for the detection of *oxa-51 *gene among *Acinetobacter baumannii* isolates. Lane 1: positive control of *oxa-51*, lanes 2- 9: positive strains of *oxa-51*.and M: 1kb DNA size marke

Sequence analysis showed that *bla*_OXA-51_ gene was detected in all isolates, 54 (83%) isolates included *bla*_OXA-23_ gene. Eight (12.5%) and 6 (9.23%) strains carried *bla*_VIM_ and *bla*_KPC_ genes respectively (Figures 1-4). Six out of eight VIM- producing strains contained both *bla*_OXA-23_ and *bla*_vim_ genes (Table 4). All of KPC producer isolates were combined by OXA-23. On the other hand, none of them contained *bla*_IMP_, *bla*_OXA-48_, *bla*_NDM-1_ and bla_SPM-1 _genes.

## Discussion

According to many studies, *A. baumannii *is known as one of the most common Gram negative bacteria that can cause nosocomial infection in health care centers especially in burned hospitalized patients (2, 3, 8). Treatment of infection due to XDR *A. baumannii *is extremely difficult and causes more morbidity and motility in hospitalized burn patients (13, 19). The results of this study indicated that 98.4% of *A. baumannii* were resistant to all tested beta lactam antibiotics and 55% of them remained susceptible just to colistin and tetracycline. Generally, carbapenemases producer strains of *A. baumannii *convert to XDR strains and can be resistant to most antibiotics used for the treatment of *A. baumannii* infections. Wounds of burned patients generally infected by multiple bacteria harboring antibiotic resistance genes located in transferable genetic elements, can be a disaster in burn care units. Resistance to all tested antibiotics in 50% of isolated KPC- producer *A. baumannii *and to all tested antibiotics except tetracycline in the remaining isolates is a significant point and creates limitation for treatment choices of this kind of infection. OXA type is the common carbapenemase in *A. baumannii *and OXA-23 is the common one in Iran (3, 8, 14, 20). Outbreaks of OXA-23 producing *A. baumannii *have been reported in several Asian countries such as China, Thailand, Taiwan (8, 6, 11). On the other hand, metallo- bata lactamase (MBL) can be considered as a second common carbapenemase in *A.* *baumannii.* In the research which was carried out by Szejbach in 2013 in Poland on MBL- producer *A.* *baumannii*, it was shown that 10.3% of isolates carried *bla*_IMP-like _gene. No *bla*_VIM-4 _was detected in the isolates (7). On the contrary, in the same study in India in 2012, 47% of isolated *A.* *baumannii *carried the *bla*_VIM_ and 0.9% of them harbored *bla*_IMP_. The variants result may be related to different ecology situation, antibiotic therapy program and variants antibiotype patterns in two different countries (21). In 2013 in China, OXA-51 gene was detected in all isolated *A. baumannii *and 94% carried *oxa-23 *gene (22). Also, the results of a study in 2012 on 68 imipenem-resistant *A. baumannii *in Iran indicated that OXA-51 and OXA-23 types were detected in all strains (20). But in the current study, 83% of isolates were identified as an OXA-23-producer which can be associated to the source of isolated bacteria. In this study, all isolates were collected from wounds of burned patients but in the study carried out in 2012 in Iran (20), the isolates were obtained from different medical specimens. On the other hand, in 2012 in another study in Iran, 88.7% of imipenem resistant A*. baumannii *carried *bla*_OXA-23__-like _gene (14) and these results are similar to ours.

In Korea in 2013, 97% of *Acinetobacter *isolates harbored the *bla*_OXA-23-like _gene (23).

Mutation in OXA type enzymes because of overuse and/or abuses of carbapenem antibiotics in clinic can lead to high carbapenem activity by OXA type carbapenemase enzymes. Because they generally have low activity of carbapenem hydrolyzing activity (9, 24). In addition, we had a report from the prevalence of OXA-48- producer *K. pneumonia* at the same time and same burn unit and this gene was located in mobile genetic elements. Thus, we have detected specifically *oxa-48* gene in isolated *A. baumannii* (25). Consequently, it can be an alarming threat in health in Iran. Moreover, as the gene of this carbapenemase is located in a mobile genetic element and can be transferred to *A. baumannii*, it can impose more complications in burn patient's therapy. In the current study, 97% of carbapemase producer strains subsequently were resistant to all beta lactam antibiotics and 80% of them were resistant to aminoglycoside. These results can be a concern for physicians because 78% of the strains were sensitive to colistin and tetracycline only. The results of this study indicated that the ability of carbapenemase production can be one of the most important reasons for the emergence of XDR *A. baumannii *in nosocomial infections. The infection is important in burned patients because their skin lose a first protective barrier, putting the patients at high risk for multiple infections.

Regarding to the mentioned above study and our results, it seems that *A. baumannii *is becoming one of the important and challengeable Gram-negative microorganism and *P. aeruginosa *is replaced by *A. baumannii* in nosocomial infection. The emergence of their XDR type makes significant therapeutic problem especially in burned patients. According to our results and another study (26), colistin remains a more effective antibiotic for the treatment of infection caused by these XDR and/or MDR microorganisms. The spread of carbapene-mase producer *A. baumannii*, as a second important pathogen that can make nosocomial infection in burned patients can be alarming for public health organization and health care systems.
